# ATPase Activity of the Subcellular Fractions of Colorectal Cancer Samples under the Action of Nicotinic Acid Adenine Dinucleotide Phosphate

**DOI:** 10.3390/biomedicines9121805

**Published:** 2021-11-30

**Authors:** Ivan Kushkevych, Mykola Bychkov, Solomiia Bychkova, Márió Gajdács, Romana Merza, Monika Vítězová

**Affiliations:** 1 Department of Experimental Biology, Faculty of Science, Masaryk University, 62500 Brno, Czech Republic; 2 Department of Therapy No 1, Medical Diagnostic and Hematology and Transfusiology of Faculty of Postgraduate Education, Danylo Halytsky Lviv National Medical University, 79010 Lviv, Ukraine; therapy.fpdo@gmail.com; 3 Department of Human and Animal Physiology, Faculty of Biology, Ivan Franko National University of Lviv, 79005 Lviv, Ukraine; solomiya.bychkova@lnu.edu.ua; 4 Department of Oral Biology and Experimental Dental Research, Faculty of Dentistry, University of Szeged, 6720 Szeged, Hungary; gajdacs.mario@stoma.szote.u-szeged.hu; 5 Faculty of Medicine, Institute of Medical Microbiology, Semmelweis University, Nagyvárad tér 4, 1089 Budapest, Hungary; 6 Department of Anesthesiology and Intensive Care, Faculty of Postgraduate Education, Danylo Halytsky Lviv National Medical University, 79010 Lviv, Ukraine; merza_romana@meduniv.lviv.ua

**Keywords:** cancer, NAADP, Ca^2+^, autophagy, acidic store, ATPase, Ca^2+^ ATPase, EPR, Ca^2+^ ATPase PM, Na^+^/K^+^ ATPase, basal ATPase activity

## Abstract

In tumor cells with defects in apoptosis, autophagy allows prolonged survival. Autophagy leads to an accumulation of damaged mitochondria by autophagosomes. An acidic environment is maintained in compartments of cells, such as autophagosomes, late endosomes, and lysosomes; these organelles belong to the “acid store” of the cells. Nicotinic acid adenine dinucleotide phosphate (NAADP) may affect the release of Ca^2+^ from these organelles and affect the activity of Ca^2+^ ATPases and other ion transport proteins. Recently, a growing amount of evidence has shown that the variations in the expression of calcium channels or pumps are associated with the occurrence, disease-presentation, and the prognosis of colorectal cancer. We hypothesized that activity of ATPases in cancer tissue is higher because of intensive energy metabolism of tumor cells. The aim of our study was to ascertain the effect of NAADP on ATPase activity on tissue samples of colorectal cancer patients’ and healthy individuals. We tested the effect of NAADP on the activity of Na^+^/K^+^ ATPase; Ca^2+^ ATPase of endoplasmic reticulum (EPR) and plasma membrane (PM) and basal ATPase activity. Patients’ colon mucus cancer samples were obtained during endoscopy from cancer and healthy areas (control) of colorectal mucosa of the same patients. Results. The mean activity of Na^+^/K^+^ pump in samples of colorectal cancer patients (*n* = 5) was 4.66 ± 1.20 μmol P_i_/mg of protein per hour, while in control samples from healthy tissues of the same patient (*n* = 5) this value was 3.88 ± 2.03 μmol P_i_/mg of protein per hour. The activity of Ca^2+^ ATPase PM in control samples was 6.42 ± 0.63 μmol P_i_/mg of protein per hour and in cancer −8.50 ± 1.40 μmol Pi/mg of protein per hour (*n* = 5 pts). The mean activity of Ca^2+^ ATPase of EPR in control samples was 7.59 ± 1.21 μmol P_i_/mg versus 7.76 ± 0.24 μmol Pi/mg in cancer (*n* = 5 pts). Basal ATPase activity was 3.19 ± 0.87 in control samples versus 4.79 ± 1.86 μmol Pi/mg in cancer (*n* = 5 pts). In cancer samples, NAADP reduced the activity of Na^+^/K^+^ ATPase by 9-times (*p* < 0.01) and the activity of Ca^2+^ ATPase EPR about 2-times (*p* < 0.05). NAADP caused a tendency to decrease the activity of Ca^2+^ ATPase of PM, but increased basal ATPase activity by 2-fold vs. the mean of this index in cancer samples without the addition of NAADP. In control samples NAADP caused only a tendency to decrease the activities of Na^+^/K^+^ ATPase and Ca^2+^ ATPase EPR, but statistically decreased the activity of Ca^2+^ ATPase of PM (*p* < 0.05). In addition, NAADP caused a strong increase in basal ATPase activity in control samples (*p* < 0.01). Conclusions: We found that the activity of Na^+^/K^+^ pump, Ca^2+^ ATPase of PM and basal ATPase activity in cancer tissues had a strong tendency to be higher than in the controls. NAADP caused a decrease in the activities of Na^+^/K^+^ ATPase and Ca^2+^ ATPase EPR in cancer samples and increased basal ATPase activity. In control samples, NAADP decreased Ca^2+^ ATPase of PM and increased basal ATPase activity. These data confirmed different roles of NAADP-sensitive “acidic store” (autophagosomes, late endosomes, and lysosomes) in control and cancer tissue, which hypothetically may be connected with autophagy role in cancer development. The effect of NAADP on decreasing the activity of Na^+^/K^+^ pump in cancer samples was the most pronounced, both numerically and statistically. Our data shows promising possibilities for the modulation of ion-transport through the membrane of cancer cells by influence on the “acidic store” (autophagosomes, late endosomes and lysosomes) as a new approach to the treatment of colorectal cancer.

## 1. Introduction

Malignant diseases constitute the second leading cause of death worldwide, and their importance is continuously increasing with the onset of the epidemiological transition and the ageing of the population globally. Colorectal cancer (CRC) is the third most commonly diagnosed type of cancer in the world and is the fourth leading cause of death from malignancy [1]. A critical role in the development of bowel cancer may be attributed to microbial communities residing in them, which produce hydrogen sulfide (H_2_S) [2,3,4,5], affecting not only the microbiota-composition of the individual, but also the health and physiology of the surrounding bowel cells [6,7,8,9].

Disruption of autophagy processes plays an important role in tumor formation [10]. Normally, autophagy is a continuous reparative process of maintaining cellular homeostasis, involving organelles, such as lysosomes and autophagosomes. These organelles belong to the so-called “acid storage” of the cells [11]. Nicotinic acid adenine dinucleotide phosphate (NAADP) may affect the release of Ca^2+^ from these organelles [12]. Apoptotic pathways are suppressed in tumor cells, and the process of autophagy further prolongs the life of atypical cells. Conversely, disorders of autophagy in normal cells may lead to their transformation into malignant cells, due to the accumulation of defective proteins. Therefore, it is important to ascertain the features and the functioning of the acid storage in both healthy individuals and patients affected by cancer, as well as the role of NAADP in their biological functions [13]. The differential diagnosis of CRC is important, as other illnesses—such as gastro-intestinal infections [14], irritable bowel syndrome, Chron’s disease, or hemorrhoids—may have a similar disease-presentation initially [15].

There is insufficient knowledge on the mechanism of action of NAADP in cancer cells, in particular, the role of NAADP in the Ca^2+^ homeostasis of cancer cells. There is a large transmembrane electrochemical gradient of Ca^2+^ between the cytosol and the intracellular environment, which leads to the entry of the Ca^2+^ ion into the cell; For the appropriate maintenance of cell signaling, it is very important to maintain a consistent, low concentration of this cation, which is achieved by the activity of Ca^2+^ pumps. Changes in plasma membrane calcium ATPase (PMCA) expression have been observed in various types of malignancies [16]. A close association was found between mutations in the Ca^2+^ ATPase EPR gene and a sharp increase in the incidence of tumors in mice [17]. The Na^+^/K^+^ ATPase may also be an interesting target for antitumor therapy [18].

As a part of this study, we aimed to determine the effect of NAADP on the activity of Ca^2+^ pumps in control and cancer samples. In addition, our investigation also aimed to answer whether the activity of the Na^+^/K^+^ pump differs in control (healthy) and colorectal cancer samples, and to evaluate the effect of NAADP on this ATPase.

Thus, the aim of our study was to determine the effect of NAADP on the energy metabolism with regard to the activity of ATPases of human colorectal cancer cells, in comparison to samples of healthy tissue of patients’ colon mucosa.

To achieve this goal, we set the following tasks:

- To estimate the effect of NAADP on the activity of Na^+^/K^+^ ATPase;
- To ascertain the effect of NAADP on the activities of Ca^2+^ ATPase of endoplasmic reticulum (EPR) and the plasma membrane (PM);
- To research the effect of NAADP on the basal ATPase activity in the patients’ colorectal cancer samples compared to the samples of healthy tissues.

The results of our study may clarify the role of the NAADP-sensitive “acid storage” (lysosomes, late endosomes, and autophagosomes) and systems of active ion transport (ATPases) in healthy and cancer cells, in addition to aiding the identification of novel targets in anti-cancer treatment.

## 2. Materials and Methods

### 2.1. Ethical Standards and Characteristics of Patients

The study was conducted according to the guidelines of the Declaration of Helsinki, and approved by the Institutional Review Board (Ethics Committee) of Department of Therapy No 1, Medical diagnostic and Hematology and Transfusiology of Faculty of Postgraduate Education, Danylo Halytsky Lviv National Medical University (protocol code No. 5, 2 September 2021).

A total of 15 patients with colorectal cancer (mean age is 58.6 ± 1.4 years) were complex examined, only 5 patients agreed to provide colorectal samples during endoscopy. The diagnosis of colorectal cancer was established according to the unified clinical protocol No 703 from 7 December 2016 on the basis of histological conclusion on the basis of morphological examination of the biopsy. Patients’ colon mucus cancer samples were obtained during endoscopy from areas affected by cancer and healthy areas (control) of colorectal mucosa of the same patients. From one patient we obtained two samples: cancer tissue and healthy tissue (control). All patients signed an informed-consent document for diagnosis and research on tissue specimens before being enrolled in the project.

### 2.2. Isolation of Subcellular Post-Mitochondrial Fraction of the Patients’ Colon Mucus

Tissue samples were collected from patients’ colons during colonoscopy. Fresh samples were washed by medium A: sucrose (250 mM), ethylene glycol tetra-acetic acid (EGTA) (1 mM), HEPES (10 mM); KH_2_PO_4_ (1 mM); pH 7.2. Then, these samples were homogenized with glass–glass homogenizer at 300 rev/min for 10 min at 0–2 °C. The homogenate was centrifuged for 10 min at 3000 *g* using a Jouan MR 1812 centrifuge (Jouan, France) to precipitate nuclei, large cells fragments, and undestroyed cells, while mitochondria remained in the supernatant 1. Next, centrifugation of this supernatant 1 was carried out for 10 min at 8500 *g* (0–2 °C). After sedimentation of the mitochondria, supernatant 2 was used for ATPase activity assay. To verify the membrane’s presence in the post-mitochondrial fraction it was centrifuged for 20 min at 15,000 *g*.

### 2.3. Assay of ATPase Activity

ATPase activity of these patients’ samples of colon mucus was estimated in post-mitochondrial subcellular fraction which was obtained by separate centrifugations as it was described previously for NK/Ly cells [18]. Briefly, tissue samples homogenized with a glass–glass homogenizer at 300 rpm for 10 min at 0 °C to 2 °C. The homogenate was centrifuged for 10 min at 3000 *g* using JouanMR 1812 centrifuge (Jouan, France) to precipitate nuclei, large cells fragments, and undestroyed cells, whereas mitochondria remained in the supernatant 1. Then, the mitochondrial fraction was sedimented by further centrifugation of this supernatant for 10 min at 8500 *g* (0–2 °C). After mitochondria sedimentation, supernatant 2 was sedimented for 20 min at 14,000 *g* and was used for ATPase activity assay. Consequently, the obtained post-mitochondrial fraction was analyzed using electronic microscopy to prove the presence of membrane vesicles, such as autophagosomes, microsomes, lysosomes, and endosomes.

ATPase activity was measured spectrophotometrically by the Fiske–Subbarow method estimating the level of inorganic phosphorus (P_i_), which is released during the ATP hydrolase reaction, and was expressed in μmol Pi/mg of protein per hour.

Briefly this post-mitochondrial subcellular fraction was transferred to a standard internal solution containing (mM) NaCI, 50.0; KCl, 100.0; Tris-HCl, 20.0; MgCl_2_, 3.0; CaCl_2_, 0.01; pH 7.0 at 37 °C. The reaction was started by adding 3 mM ATP (Sigma, Burlington, MA, USA). NAADP (Sigma, USA) in concentrations 1 μM was added to incubation suspension for estimating its effect on ATPase activity.

- The activity of Na^+^/K^+^ ATPase was expressed as the difference of P_i_ in the medium with or without ouabain (Sigma, USA). The samples (without ouabain) instead contained incubation medium in equal measure. Basal Mg^2+^ ATPase activity was determined in an incubation medium that did not contain CaCl_2_, but included EGTA, as well as ouabain. Thapsigargin (Sigma, USA) was added to inhibit the activity of the Ca^2+^ ATP of EPR. The activity of Ca^2+^ ATP of EPR was also calculated as the difference of P_i_ in the medium with inhibitors (thapsigargin and ouabain) or without these compounds. The samples (without thapsigargin and ouabain) contained incubation medium in equal measure;
- Thapsigargin and ouabain were previously dissolved in DMSO in a separate aliquot, then dissolved again in another aliquot in internal solution and then was added to the incubation medium in concentration 1 μmol. Other samples (without thapsigargin and ouabain) instead contained incubation medium in equal measure.

NAADP (1 μM) (Sigma, USA) was added to the incubation medium to determine its effect on ATPase activity. ATPase activity was expressed as micromoles of inorganic phosphorus, equivalent to 1 mg of protein per 1 h (μmol P_i_/mg of protein per hour).

### 2.4. Calculation of Specific ATPase Activity

The total ATPase activity of the post-mitochondrial fraction of colon mucous was calculated by the difference of inorganic phosphorus in the media with different compositions: (a) specific Na^+^/K^+^ ATPase activity was calculated as difference of inorganic phosphorus content in medium with or without ouabain (1 mM); (b) for the determination of Ca^2+^/Mg^2+^ ATPase activity, the difference between the total Ca^2+^/Mg^2+^ and Na^+^/K^+^-ATPase activity was quantified (c) thapsigargin was used to calculate SERCA (sarco/endoplasmic reticulum Ca^2+^ ATPase) contribution into the total Ca^2+^/Mg^2+^ ATPase activity (d) specific basal Mg^2+^ ATPase activity was determined in an incubation medium that contained 1 mM EGTA and lacked ouabain. In all experiments, the incubation medium was used as a control for the enzymatic ATP hydrolysis.

### 2.5. Statistical Analysis

We calculated such statistical indexes as M is mean and m is standard error of the mean. The data in text are expressed as mean ± SEM. We used the Kolmogorov–Smirnov test (K–S test) for verifying compliance with parametric (normal) distribution. The significance of differences between experimental groups was calculated using Wilcoxon–Mann–Whitney, when the data distributions were not normal, while Student’s *t*-test was used when data distribution was normal. Statistical processing of information was performed using the Software SPSS Statistics 17.0 (SPSS, Inc., Chicago, IL, USA) *p* ˂ 0.05 was considered to be statistically significant.

## 3. Results

### 3.1. Evaluation of the Effect of NAADP on the Activity of Na^+^/K^+^ ATPase of Post-Mitochondrial Fraction of Human Colorectal Cancer Tissue Samples vs. Normal Tissue

**Figure 1:**
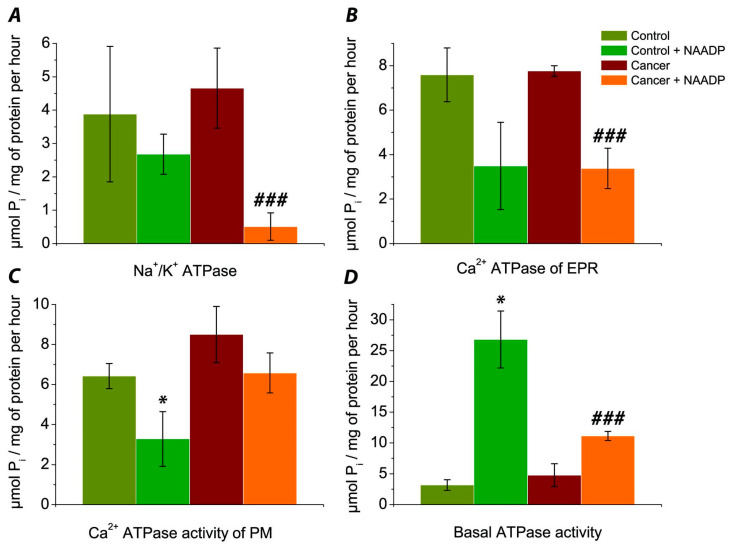
Comparison of NAADP effect on ATPase activity (μmol P_i_/mg of protein per hour) in colorectal mucosa samples from healthy area of patients’ mucosa (control) and the samples of colorectal tumor’s area of the same patients (cancer): Na^+^/K^+^ ATPase activities, *n* = 5 pts. (5 samples of control vs. 5 cancer samples) (**A**); Ca^2+^ ATPase of EPR, *n* = 5 pts. (5 samples of control vs. 5 cancer samples) (**B**); Ca^2^^+^ ATPase activity of PM, *n* = 5 pts. (4 samples of control vs. 5 cancer samples) (**C**); Basal ATPase activity, *n* = 5 pts. (5 samples of control vs. 5 cancer samples) (**D**). * indicates *p <* 0.05 (vs. control); ### is *p* < 0.01 (vs. cancer). The data are expressed as mean ± standard error of the mean.

The mean activity of Na^+^/K^+^ ATPase of post-mitochondrial fraction in colorectal cancer patients’ (pts) samples was 4.66 ± 1.20 μmol P_i_/mg of protein per hour (*n* = 5 pts). In control samples (from healthy colorectal mucosa tissues) of the same patient (*n* = 5 pts) this value was 3.88 ± 2.03 μmol P_i_/mg of protein per hour. Thus the activity of Na^+^/K^+^ ATPase was approximately 20% higher in cancer samples compared to their control counterparts (Figure 1A), but this increase was not statistically significant.

When NAADP was added to the control medium of incubation of these samples, the mean activity of Na^+^/K^+^ ATPase was 2.68 ± 0.60 μmol P_i_/mg of protein per hour (*n* = 5 pts). Thus, NAADP reduced the activity of Na^+^/K^+^ ATPase in control, but this effect was not statistically significant (*p* > 0.05). In cancer samples, adding NAADP extremally decreased the mean activity of Na^+^/K^+^ ATPase to 0.51 ± 0.41 μmol P_i_/mg of protein per hour (Figure 1A). Therefore, NAADP statistically decreased the activity of Na^+^/K^+^ ATPase by 9-times in cancer samples (*p* < 0.01). Hence, the effect of NAADP on inhibition of the activity of Na^+^/K^+^ pump was more effective in cancer tissue.

### 3.2. Determination of the Effect of NAADP on the Activity of Ca^2+^ ATPase EPR of the Post-Mitochondrial Fraction of Colorectal Cancer Tissue Samples vs. Normal Tissue

In control patients’ tissue samples, the activity of Ca^2+^ ATPase EPR of the post-mitochondrial fraction of colon mucosa showed a mean of 7.59 ± 1.21 μmol P_i_/mg of protein per hour (*n* = 5 pts). In cancer samples, the activity of Ca^2+^ ATPase EPR varied from 7.00 to 8.26, with a mean value of 7.76 ± 0.24 μmol P_i_/mg of protein per hour (*n* = 5 pts), which is only slightly higher than in samples from control tissues (Figure 1B). When NAADP was added to the control, the activity of Ca^2+^ ATPase EPR amounted to 3.49 ± 1.96 μmol P_i_/mg of protein per hour (*n* = 5 pts). Thus, NAADP showed the tendency to decrease the activity of Ca^2+^ ATPase EPR in control. Under the influence of NAADP on the subcellular fraction of cancer samples, the mean activity of Ca^2+^ ATPase EPR was 3.38 ± 0.91 μmol P_i_/mg of protein per hour (*n* = 5 pts). This decreasing was statistically significant (*p* < 0.01).

Thus, NAADP reduced the activity of Ca^2+^ ATPase EPR in both control and cancer cells by about 2-times (Figure 1B), but this effect was statistically significant only in cancer samples.

### 3.3. The Effect of NAADP on the Activity of Ca^2+^ ATPase PM of the Post-Mitochondrial Fraction of Colorectal Cancer Cells vs. Normal Tissue

Activity of Ca^2+^ ATPase of PM in control samples was 6.42 ± 0.63 μmol P_i_/mg of protein per hour (*n* = 4 pts). Unfortunately we have only 4 control samples, because one was lost during the experiments. In samples of colorectal cancer tissue, the activity of Ca^2+^ ATPase of PM was 8.50 ± 1.40 μmol P_i_/mg of protein per hour (*n* = 5 pts). Thus the activity of Ca^2+^ ATPase of PM had a tendency to be higher in cancer samples comparatively to control tissue (Figure 1C). Incubation the of post-mitochondrial fraction of control samples with NAADP caused a statistically significant (*p* < 0.05) decrease in the activity of Ca^2+^ ATPase of PM by approximately 2 fold to 3.28 ± 1.36 mol P_i_/mg of protein per hour (*n* = 4 pts). Adding NAADP to the post-mitochondrial fraction of colorectal cancer samples decreased the activity of this pump to 6.58 ± 1.00 μmol P_i_/mg of protein per hour (*n* = 5 pts), but this effect was not statistically significant.

Thus, NAADP statistically decreased the activity of Ca^2+^ ATPase of PM in control samples (Figure 1C) and showed the tendency to decrease this index in cancer samples as well.

### 3.4. Comparison of Basal ATPase Activity without Exposure and under the Action of NAADP

In control samples of patients’ colorectal mucosa (Figure 1D), the basal ATPase activity ranged from 0.42 to 5.88, the mean was 3.19 ± 0.87 μmol P_i_/mg of protein per hour (*n* = 5 pts). Basal ATPase activity of colorectal cancer samples ranged from 2.10 to 9.66, with a mean of 4.79 ± 1.86 μmol P_i_/mg of protein per hour (*n* = 5 pts). So, a tendency to increase the basal ATPase activity in cancer vs. control was observed. After NAADP-treatment of control samples, basal ATPase activity increased significantly and showed 26.80 ± 4.62 μmol P_i_/mg of protein per hour (*n* = 5 pts). Thus, NAADP caused 8-fold, statistically significant increases of this index (*p* ˂ 0.01) vs. control. When NAADP was added to the incubation medium of cancer samples, the mean of basal ATPase activity was 11.13 ± 0.74 μmol P_i_/mg of protein per hour (*n* = 5 pts), which was 2-fold higher vs. mean of this index in cancer samples without NAADP treatment.

Thus, NAADP caused a strong increase in basal ATPase activity by 8-folds in control samples and only a 2-fold increase in this index in the control.

## 4. Discussion

We hypothesized that the activity of ATPase in cancer tissue is higher than in healthy tissue, due to the intensive energy metabolism of tumor cells, which is a prerequisite for their continuous cell division and proliferation [19]. Our results confirmed this assumption: the activity of Na^+^/K^+^ pump and Ca^2+^ ATPase of PM in cancer tissues was higher than in healthy ones by 17% and 24%, respectively, but this increase was not statistically significant (*p* > 0.05). Activity of Ca^2+^ ATPase of EPR and basal ATPase activity did not have very significant changes in cancer tissue, in comparison to control samples. This may be explained by the fact that Ca^2+^ ATPase of EPR is often inhibited in cancer, because SERCA inhibition is thought to promote cell survival [20].

The Na^+^/K^+^ pump is a transmembrane protein complex that maintains the ionic and osmotic balance of the cells of higher eukaryotes. This ATPase is a universal transducer and integrator of cellular signals, and it plays a key role in cell adhesion [12]. Abnormal expression of this protein is associated with the development and progression of various types of malignancies. It has been shown that the expression of α- and β-subunits of Na^+^/K^+^ ATPase of cancer and normal cells differs considerably, thus creating a powerful diagnostic tool and target for the action of inhibitors and ligands of the sodium-potassium pump [17].

We found that the activity of this pump in cancer tissues was about 20% higher than in control samples, but this increase was not statistically significant. However, observed tendency to increasing the activity of Na^+^/K^+^ ATPase in cancer is in agreement with findings of other authors, showing that the baseline expression and activity of Na^+^/K^+^ ATPase were enhanced in breast cancer, compared to a non-tumorigenic breast cell line [21], in patients with ovarian serous cystadenocarcinoma [22]. In samples of patients’ colorectal cancer, highly expressed Na^+^/K^+^ ATPase isozymes were found [23]. We established a statistically significant decrease in Na^+^/K^+^ ATPase activity under the action of NAADP in cancer samples and strong tendency to reduced this pump in control: NAADP reduced the activity of Na^+^/K^+^ ATPase in colorectal cancer samples by 9.1 times. This result is similar to our previous findings regarding the effects of NAADP, studied on murine NK/Ly cells [19], as well as in a model system consisting of rat liver tissue [24].

This effect of NAADP may be explained by a powerful release of Ca^2+^ from the acidic stores by NAADP, causing an inhibitory effect on Na^+^/K^+^ ATPase activity. For example, such an inhibitory effect of higher intracellular Ca^2+^ on the sodium pump was shown for human red blood cells [25]. Another possible explanation may be related to the pH change caused by the effects of NAADP. A.J. Morgan et al. estimated that NAADP caused alkalization of the endo-lysosomal lumen [26]. Thus, it is also likely that NAADP decreased activity of Na^+^/K^+^ ATPase due to change in pH. Alkalization of the biological environment to higher than pH 7.5 inhibits this protein, as well as the process of acidification to pH values lower than 6.0 [27].

In our case, NAADP indirectly caused an inhibitory effect on Na^+^/K^+^ ATPase activity. It also was shown that inhibitors of Na^+^/K^+^ ATPase exhibit antitumor effects on hepatocellular carcinoma models [28]. The study of Trenti and co-authors [29] showed that inhibition of Na^+^/K^+^ ATPase promotes autophagy and causes an anticancer effect. The hypothetical role of autophagy in this process was confirmed by our experiments which use NAADP as a tool to target acidic stores (autophagosomes, late endosomes, and lysosomes) and indirectly inhibit Na^+^/K^+^ ATPase. Thus, this ATPase may present as a potent target in anticancer treatment.

Ca^2+^ ATPases are a key factor in maintaining the asymmetric distribution of calcium ions between different cell compartments. Remodeling of Ca^2+^ signaling is an important step in cancer progression. The expression of Ca^2+^ pumps is highly regulated in breast cancer cells [30]. Increased gene expression of this protein has been found in various types of cancers [15]. Changes in PMCA-expression and related changes in the release of calcium from the cell in colon cancer cells contribute to cancer growth [31].

We found that NAADP caused a tendency to decrease the activity of Ca^2+^ ATPase of PM in patients’ colorectal cancer tissue, but in control samples, this effect was more pronounced. NAADP caused statistically significant reduction in the activity of Ca^2+^ ATPase of PM by 2 folds. Some studies suggest that PMCA4 affects the colon cancer cell growth cycle and cell death. PMCA4 is overly expressed in higher differentiated human colon cancer samples and HT-29, Caco-2 cells. This suggests that PMCA4 affects colorectal cancer cell death [32]. PMCA4-knockout cells are able to avoid the decrease in cellular viability, and cause a lack of sensitivity to apoptotic stimuli [33].

Ca^2+^ ATPases of EPR pump Ca^2+^ into the endoplasmic reticulum lumen modulating cytosolic Ca^2+^ concentrations to regulate various cellular processes including cell growth. A downregulation of SERCA3 protein expression was reported in gastric and colon cancer cell lines and showed that in vitro cell differentiation increases its expression [30]. Here, it was estimated that NAADP showed the tendency to decrease the activity of Ca^2+^ ATPase EPR in control but it statistically reduced the activity of Ca^2+^ ATPase of EPR in cancer samples (*p* < 0.01). We suppose that NAADP decreased Ca^2+^ ATPase activity due to releasing Ca^2+^ from the “acid storage”.

Our data are consistent with previous findings observed on murine NK/Ly cells: NAADP decreased activities of Ca^2+^ ATPase of EPR and basal Mg^2+^ ATPase [19]. Recently Pawan Faris et al. have shown that NAADP induces intracellular Ca^2+^ release through the two-pore channel TPC1 in metastatic colorectal cancer cells [34].

We observed different effects of NAADP on Ca^2+^ ATPase EPR and PM in control and colorectal cancer patients’ samples. These data confirmed distinctive roles of NAADP-sensitive “acidic store” (autophagosomes, late endosomes, and lysosomes) in control (healthy) and cancer tissue, which hypothetically may be associated with the role of autophagy in cancer development.

We also observed an extremely large increase in (8.4 fold) the activity of basal Mg^2+^ ATPase by NAADP in control colon tissue samples. This agrees with our previous findings on rat’s liver tissue [24]. This effect is not associated with the increasing Ca^2+^ concentration because of the presence of EGTA in the incubation medium. We explain such effects with changes of pH. S. O. Kosterin et al. [27] showed that H^+^ is a competitive inhibitor of a given enzyme: the increase in proton concentration leads to a decrease in the affinity of Mg^2+^ ATP substrate. Thus NAADP not only releases Ca^2+^ from acidic stores, but simultaneously changes pH in subcellular fraction of human colorectal cancer, probably due to a Ca^2+^-H^+^-exchanger in membranes of these organelles. In cancer tissue, this effect of NAADP on activity of basal Mg^2+^ ATPase was not as pronounced (only a 2 fold increase). This probably indicates a different role for NAADP-sensitive acidic storages in cancer cells.

Thus, the obtained data show promising possibilities for the modulation of ion-transport through the membrane of cancer cells by influence on “acidic store” (autophagosomes, late endosomes, and lysosomes) as a potentially new approach to the treatment of colorectal cancer. These results expand the understanding of the mechanisms of cancer development and the role of NAADP-sensitive acid stores in this process and also highlight possible therapeutic targets for cancer treatment.

## 5. Conclusions

During our study, we have found that the activity of Na^+^/K^+^ pump and Ca^2+^ ATPase of PM in cancer tissues had a tendency to be higher than in control. Activity of the calcium ATPase of EPR and basal ATPase activity were not very intensively changed in cancer tissue comparison to control. NAADP causes a decrease in the activities of Na^+^/K^+^ ATPase, Ca^2+^ ATPase EPR, and Ca^2+^ ATPase of PM in both control and cancer samples, except basal ATPase activity, which was increased by NAADP. The effect of NAADP on decreasing the activity of Na^+^/K^+^ pump in cancer samples was especially pronounced. Thus, based on our experimental results, NAADP modulates ATPase activity in cancer tissues and can decrease the energetic status of tumor cells. This makes NAADP a potential tool in the future of cancer treatment.

## Data Availability

Not applicable.
